# Continued Neurogenesis in Adult *Drosophila* as a Mechanism for Recruiting Environmental Cue-Dependent Variants

**DOI:** 10.1371/journal.pone.0002395

**Published:** 2008-06-11

**Authors:** Selim Ben Rokia-Mille, Sylvette Tinette, Gilbert Engler, Laury Arthaud, Sophie Tares, Alain Robichon

**Affiliations:** Department of Biology and Biotic Interactions, CNRS/INRA, University Nice Sophia-Antipolis, Sophia Antipolis, France; Temasek Life Sciences Laboratory, Singapore

## Abstract

**Background:**

The skills used by winged insects to explore their environment are strongly dependent upon the integration of neurosensory information comprising visual, acoustic and olfactory signals. The neuronal architecture of the wing contains a vast array of different sensors which might convey information to the brain in order to guide the trajectories during flight. In *Drosophila*, the wing sensory cells are either chemoreceptors or mechanoreceptors and some of these sensors have as yet unknown functions. The axons of these two functionally distinct types of neurons are entangled, generating a single nerve. This simple and accessible coincidental signaling circuitry in *Drosophila* constitutes an excellent model system to investigate the developmental variability in relation to natural behavioral polymorphisms.

**Methodology/Principal Findings:**

A fluorescent marker was generated in neurons at all stages of the *Drosophila* life cycle using a highly efficient and controlled genetic recombination system that can be induced in dividing precursor cells (*MARCM* system, *flybase* web site). It allows fluorescent signals in axons only when the neuroblasts and/or neuronal cell precursors like SOP (sensory organ precursors) undergo division during the precedent steps. We first show that a robust neurogenesis continues in the wing after the adults emerge from the pupae followed by an extensive axonal growth. Arguments are presented to suggest that this wing neurogenesis in the newborn adult flies was influenced by genetic determinants such as the frequency dependent *for* gene and by environmental cues such as population density.

**Conclusions:**

We demonstrate that the neuronal architecture in the adult *Drosophila* wing is unfinished when the flies emerge from their pupae. This unexpected developmental step might be crucial for generating non-heritable variants and phenotypic plasticity. This might therefore constitute an advantage in an unstable ecological system and explain much regarding the ability of *Drosophila* to robustly adapt to their environment.

## Introduction

Drosophila survival depends on their capacity to exit a niche that has become unfavorable and/or hostile and find alternative locations where food resources are more abundant. The efficiency of this exploration depends upon the simultaneous integration of neurosensory signals that are used to guide flight trajectories [1], [2]. This implies that robust sensory systems are simultaneously in operation, such as vision adapted to moving objects during flight and olfaction for odorant detection in air currents. Mechanoreceptor feedback also acts in combination with the vision and olfaction systems to change the flight direction by rapid stereotyped turns, which characterizes flight behaviour in Drosophila [3].

Fly wings appear to assume many roles such as taste, touch perception, propioception, courtship singing, in addition to flight. However, the physiological relevance of some wing sensory cells are poorly understood (for example, the presence of taste organs on the wing). Intriguingly, a spatially restricted expression of taste receptors has been described for the wing [4] and, surprisingly, a large gene family of odorant binding proteins is expressed not only in olfactory organs as expected, but also in wing gustatory sensilla [5]. Some isoforms of this gene are exclusively expressed in taste organs including the taste bristle of the wing [5]. Many lines of evidence using in situ hybridization methodologies have confirmed that a chemosensory gene family encodes both odorant and taste receptors [6]. This suggests overlapping roles and functions between olfactory and gustatory organs in *Drosophila* or at least a diffuse physiological frontier between both systems.

To our knowledge, no report has demonstrated in any insect how mechanosensory cues encoded from wing neurons are integrated by the nervous system for flight guidance. However, we have observed previously that an unilateral lesion of the costal nerve still allows the fly to take off but abolishes the directional flight [7]. The sensory structures in the *Drosophila* wing present some major advantages for use as a model system to investigate the dispersion behavior of insects. The first of these is that they are highly tractable for fluorescent marker based analysis. Second, these systems have been intensively studied to date. Briefly, two major neuronal cell types co-exist: the mechanoreceptors which are sensors of touch perception and proprioception [8] and the chemoreceptors which express odor-binding proteins but without any clearly demonstrated function to date [9], [5]. The two types of sensory information that are processed by these receptor types are simultaneously transported via the same nerve.

The lineage of the neurons that constitute the mechanosensory and chemosensory bristles on the wing or notum of *Drosophila* has been well characterized [10]–[14]. Some of the sensory hairs (slender) on the anterior wing margin are chemoreceptors that express odor-binding proteins. The stout bristles of the anterior margin, however, are mechanoreceptors. Bristle sensilla are also located along the anterior wing margin and large campaniform sensilla are found along the wing median vein (vein III). Briefly, a sensory organ precursor (SOP) initially divides asymmetrically to produce pIIa and pIIb cells. PIIa divides to provide a posterior socket cell and an anterior hair cell. PIIb divides to provide pIIIb and a glial cell. Finally, pIIIb divides to produce a sheath cell and a neuron (see [12] for review). The mechanoreceptors comprise a single unique neuron whereas the chemoreceptors consist of a cluster of 5–6 neurons [11]. Each division from the SOP is therefore asymmetric and generates non-neuronal cells such as posterior socket cells or anterior hair cells [15]–[20]. The three types of cell harboring the genetic constructs we utilized in our current study (pIIb, pIIIb, and mature neurons) would be expected to show fluorescence because they express *elav* (In the *MARCM* system, the *GFP* molecules are under the control of the *elav* promoter; see *supplementary material*, figure S1) [21]. Finally each of the larval sensilla degenerate during metamorphosis and the generally accepted view is that the precursors of the adult sensilla appear during the late third larval instar [10]–[14].

The *Drosophila* wing is also an excellent system to verify whether behavioral variants harbor subtle differences in their neuronal circuitry, and we speculated whether a natural polymorphism repertoire exists that is used by this species as an adaptive tool to respond to environmental changes. The scarcity of food resources or a high population density triggers dispersion and colonization of new spaces. It is possible that these environmental conditions regulate and/or control epigenetic mechanisms, as suggested in many previous studies in *Drosophila* [22]–[27].

To further elucidate these phenomena, we reasoned that the most effective approach would be to use a genetic tool which allows us to visualize the process of neurogenesis during the life cycle of *Drosophila*. The technique used in our present study to achieve this is based on mitotic recombination induced by the enzyme ‘*flipase’* under the control of a heat shock promoter. A fluorescent marker is generated in neurons in which genetic recombination was previously induced in the dividing precursor cells. This drives fluorescence at any stage of the *Drosophila* life cycle in mature neurons, but only when their precursors undergo division at the time of induction. In other words, there is no fluorescence in mature neurons in the absence of recombination events in the progenitor cells (this system is publicly available at *Bloomington Center Indiana University US* and was donated by Liqun *Luo* and co-workers). Because the recombination in this system is induced, we aimed to observe whether adult *Drosophila* undergo neurogenesis and if so, how environmental cues may affect this process.

From our initial data, we observed robust neurogenesis in the adult wing after emergence from the pupa, which cannot be accounted for simply by a residual completion at the very end of the developmental process. These unexpected results have not been obtained previously because the adult wing cuticle is extremely resistant to any of the treatments used in immunohistology studies.

We then considered the evolutionary advantage of this system and speculated as to why the process of natural selection did not lead to the completion of neurogenesis at the end of the pupal stage, as is commonly accepted. To approach this issue, we investigated the variability of wing neuronal development when *Drosophila* flies are placed under different environmental conditions. To this end, we took advantage of a well established model whereby exploratory or sedentary larval behaviors are under the control of a single gene [28]–[30]. This gene (*for*) encodes a cGMP-dependent kinase and controls foraging behavior in order to adapt the flies to their environmental conditions. High density animal rearing conditions boost the larval exploratory phenotype over successive generations, whereas the opposite low density conditions favor the sedentary phenotype. Interestingly, in adult *Drosophila*, some of the expressed *for* alleles induce fidelity to the site of birth, whereas others induce a predilection to explore new habitats and engage in foraging [31]. This is therefore a rare case of a natural behavior polymorphism that is maintained by the frequency dependent selection of only one gene [32]–[35]. This prompted us to investigate whether the expression of alleles of the well-known *for* gene in *Drosophila* that confer *Rover* behavior might influence neurogenesis in adult wings.

We were also interested in analyzing the link between adult neurogenesis and the complex gene networks that are involved in larval development. We thus investigated how the pleiotropic disorders generated during the larval stages by environmental stresses such as food scarcity might interfere with adult neurogenesis when the newly born animals have access to optimal food conditions. Valuable insight was previously gained from normally-proportioned but small adults (definitive reduction in body size) when the larvae were bred under poor nutritional conditions [22]–[26], [36 for review]. These newborn adults were found to be able to revert to normal size in succeeding generations that were placed in optimal food conditions [22]–[26], [36]. The completion of wing neurogenesis at the end of pupa stage would likely preclude any chance of adult fly survival if the larvae faced environmental stress. Conversely, we reasoned that unfinished neurogenesis might allow newborn animals that had experienced stress as larvae, to “jump” to a nearby source of nutritious food and to complete their neuronal development. We reasonned that newly born flies are capable of awkward displacement by poorly controlled flight on short distance which should increase the chance of ramdom discovering of food source. Alternatively the transfer of newly born adults to the next fruit might be partially guided depending on the degree of completion of olfactory and visual systems. Hence, we have attempted in our present study to determine whether incomplete neurogenesis in newborn flies constitutes an adaptation mechanism in fluctuating and unstable environments. This phenomenon allows flies that are newly born after enduring poor nutritional conditions during larval stages to rescue their wing neuronal architecture and complete their development if they are able to find nutrients in close proximity. This stretched development process might constitute a broad mechanism selected by evolution to correct flaws inflicted by unfavorable environmental conditions in precedent stages of growth.

We analyzed then whether fluctuating environmental conditions (food resources *versus* population density) might influence the neuronal wing development in *Drosophila* in combination with genes such as the frequency-dependent alleles of *for*. Because the pupa stage in *Drosophila* lasts 5 days, during which time environmental conditions might change drastically, incomplete neurogenesis in the newborn adult wings might constitute a time window for plasticity influenced by heritable traits. Breeding conditions in the laboratory provide insights into how natural fluctuating environments might affect the phenotypic outcome. The effects of population density were investigated to determine whether non heritable variants involving wing neuronal components are recruited. Strictly *Rover* or *sitter* mutants were also analyzed in this context. Overall, the genetic tools employed in this study allowed us to analyze how environmental cues can shape an unfinished neuronal architecture in insects. This methodology thus turned out to be a useful approach to addressing some of the challenging questions about how this overall system works. To our knowledge little is known about adult wing neurogenesis in insects. Neurogenesis in adult *Drosophila* brain has never been reported. However, adult neurogenesis is intense in brain of some insects like crickets. Adult crickets reared in an enriched sensory environment present a stronger neurogenesis as compared with crickets reared in an impoverished environment [37]. Moreover, the exposure of odours stimulates the neuroblast proliferation in mouse and the outcome of newborn mature neurons in the adult olfactory bulb [38]. Adult neurogenesis in brain in mammals or birds seems to play a role in establishing a temporal order of events by clearing out old traces of experiences and consolidating new memory [39]. Many reports suggest that environmental cues are able to influence adult neurogenesis and newly born neurons participate to learning, odours integration and adaptation to environment [37]. To our knowledge, the plasticity of neurosensory cells in insect wing has never been described. In this report, we expose few elements. i) first, we observed a novel neurogenesis pattern in *Drosophila* adult wing after emergence from pupae. ii) We hypothesized that this may be an adaptation to fluctuating environment, so that when larvae suffer from poor food conditions, unfinished wing neurogenesis in newly born adults might constitute a second chance if they encounter optimal food conditions. We investigated also whether this late neurogenesis might be waiting for some epigenetic events depending on environmental conditions. iii) We examined whether a well-described natural genetic polymorphism that affects exploratory behaviour was correlated with wing neurogenesis patterns. iv) We investigated whether population density, as a cue for whether dispersal should be favoured, influences wing neurogenesis patterns. v) We examined finally whether exploratory behavior in conditions where flies are allowed only to walk, is affected by lesioning the wing costal nerve in which the neurogenesis occurs. This set of experiments was performed in order to propose some putative functions of the wing late neurogenesis. We advance arguments on the evolutionary advantages of uncompleted wing neuronal network at the end of pupae stage.

## Results

### Evidence for a continued process of maturation in the sensory organs of the Drosophila adult wing

We first investigated whether adult *Drosophila* wings undergo neurogenesis, which would imply the presence of dividing precursor neuronal cells *in situ.* The method used in this analysis (the *MARCM* system) was originally described by Lee and Luo and is based upon a repressible neuronal cell marker and a mitotic recombination enzyme under the control of a heat-shock promoter [40]. This technique facilitates the induction of a fluorescent marker in axons at any stage of the fly life cycle, but with the condition that the neuroblasts and/or precursors must first undergo cell division. The recombination event is triggered by the flipase enzyme which is expressed under the control of a heat-shock promoter that physically separates the *Gal4* transcriptional activator or the *Gal80* inhibitor in each sibling cell (see Figure S1) [40]. Controls without heat shock are shown in Figure S2.

**Figure 1:**
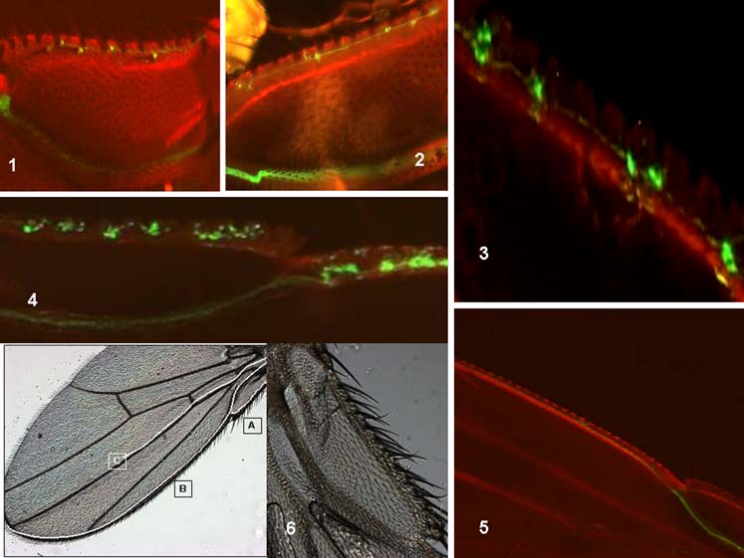
Recombination induced at the early pupal stage: fluorescence analysis in the Drosophila wing after adult emergence. *Analysis of fluorescence in the proximal fork of the anterior wing margin of the adult fly:* Progenies of the A+B cross (see *Materials and Methods*) were heat-shocked at the early pupal stage and the adult wings were analyzed for GFP fluorescence. The images shown represent the proximal part of the wing (see part 6 in photo). The anterior wing margin bifurcates in two directions. The inner branch drives the bundle of axons toward the thoracic structures. The wing structures (margin and veins) appear in red. (1) Tiny fluorescent cells appear first. (2) Tiny doublets indicating cell division are evident and axons appear to elongate in a “pioneer tract” (one day after emergence). (3) Heterogeneous spots comprising one, two or four labeled cells are visible. (4) Homogeneous clusters comprising one highly-labeled cell and a string of weakly-labeled cells are visible. The wings samples indicated in panels (3) and (4) are about 5 hours old. (5) Wing from a 5 day old adult. An axonal bundle is evident, as is the absence of cell body labeling. (6) Representative photograph *of a Drosophila* wing shows the three neuronal paths (A,B,C) and the proximal part of the wing.

The labeled adult wings obtained from flies in which mitotic recombination was induced at an early pupal stage clearly show that the neuronal cells are organized according to stereotyped paths: the anterior wing margin (vein 1) and the median path (vein 3). The fluorescence in the proximal fork of the anterior wing margin was first analyzed and very small fluorescent cells were found initially in the adult wing. The appearance of doublets was then distinctly observed, which suggested binary cell division (Figure 1). In other cases, we observed intense fluorescence in clusters of 4 cells, in doublets or single cells in the same wing, when the fly had aged by a few hours (Figure 1). Intense fluorescence was also seen in one particular cell within clusters, again suggesting cell division (Figure 1). Because each fluorescence spot is supposed to be a trace of an initial sensory organ precursor (SOP), these data argue in favor of a heterogeneous process of neuronal division in the adult *Drosophila* wing before the sensory organs are completed. The variability of labeling in the proximal fork between individuals at the same age after birth is shown in Figure S3.

**Figure 2:**
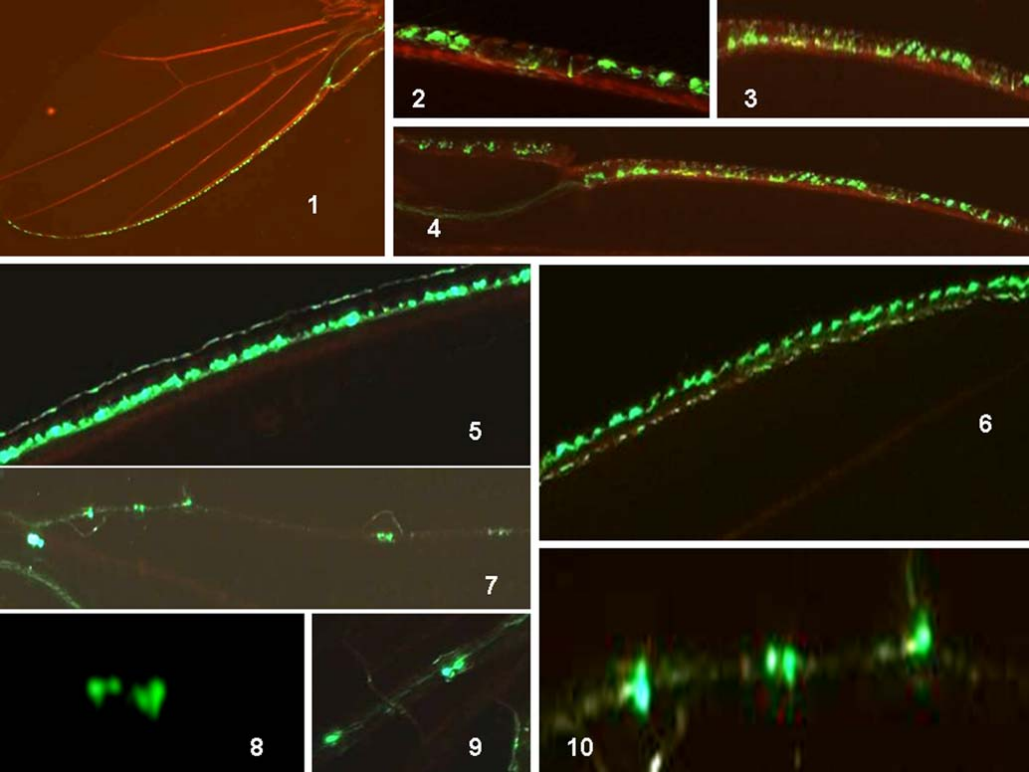
Recombination induced at the early pupal stage: fluorescence analysis in the Drosophila wing after adult emergence. *Analysis of fluorescence in the anterior wing margin after emergence of the adult fly (1–6):* Progenies of the A+B cross (see *Materials and Methods*) were heat-shocked at the early pupal stage and the adult wings were analyzed for GFP fluorescence. (1–4) Representative images of two typical wings are shown. Note the alignment of the fluorescent cells, and that there are no axons visible as yet (alignment of “boutons”). Note also the disparity between differentiation processes in the same wing. (5) Heavily fluorescent dorsal row of sensilla along the wing margin. (6) Another typical wing shows two dorsal rows of fluorescence: one is intense, the other weak. The latter weak row does not exist in (5), although the wings are of roughly the same age. (see photo 1 in this panel and figure 1 photo 6 for dimensions and orientation). *Analysis of fluorescence in the median vein III of the adult wing at between 5 and 10 hours after emergence (7–10):* (7–10) Campaniform sensilla along vein III of an adult wing. One sensilla has four labeled cells, another shows two labeled cells, and two other sensilla show one labeled cell undergoing division (planar anaphase). (see photo 1 in this panel and figure 1 photo 6 for dimensions and orientation).

We next analyzed the fluorescent signals in the anterior wing margin (vein 1). After their emergence, the labeled cells were readily identifiable and projections were absent (Figure 2). A dorsal row of cells was found to be intensely fluorescent. Interestingly, some flies showed a second dorsal row of weakly fluorescent cells, which once again suggests cell division (Figure 2). Finally, the fluorescence in the adult wing along the median vein (vein 3) was analyzed (Figure 2). Doublets of labeled cells and single labeled cells were clearly seen. A more refined analysis showed that four labeled cells co-exist in the same sensilla. We obtained many flies in which spots of fluorescence consisting of four cells, two cells or single cells entering anaphase (yellow protruding spot), co-existed in the same wing. These differing labeled patterns were also found randomly in our test population, such that none of the young flies seems to be identical.

Because each of the fluorescent spots will eventually become mature campaniform sensilla, our data suggest that continued active division and maturation of the sensory organ occurs after the emergence of the adult and that this is a weakly synchronized process. In conclusion, we observed variations in the same wing, between the two wings of the same fly, and between flies of the same age in terms of the maturation process of the mechano/chemosensory organs (see also Figure S3). These variations observed between the emerged flies in terms of their wing neuronal development were also observed using an additional construct expressing an hybrid synaptotagmin-green fluorescent protein (*GFP-syt)* in the neurosecretory vesicles (see Figure S4).

### The growth of axons and axonal bundle formation occurs in the adult wing

**Figure 3:**
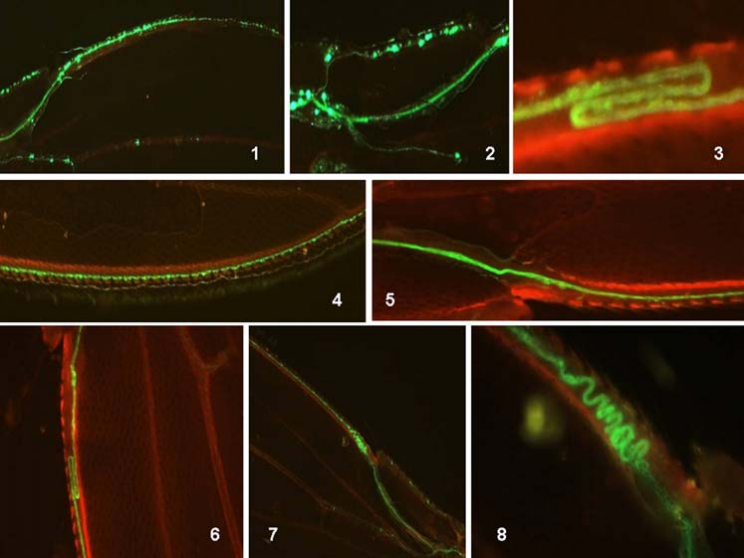
Analysis of fluorescence in the anterior wing margin of the adult fly at 5 days after emergence from the pupa (mitotic recombination was induced at an early pupal stage). (1,2) Representative images of wings at 1 day after emergence for comparison. Labeled sensilla in the external branch (fork) of the wing margin and a strongly labeled axonal bundle in the proximal wing are present. Grape-like groups of cell attached to the bundle of axons, suggest recent neuronal differentiation events. (3,4) 5 day old wings. The absence of labeled cell bodies and clusters but a strongly fluorescent axonal bundle is observed. (5–8) “Mistakes” in the pattern of fluorescence in 5 day old wings. Bundles of axons along the anterior wing margin and the absence of labeled cell bodies are observed. (5,6) Hairpin-like axonal bundle. (6,7) “Errors” in the axonal bundle showing zigzag path-finding at the proximal part of the anterior wing margin. See photo 6 in figure 1 for dimensions and orientation.

After day one, the projections in the *Drosophila* wing margin first start to fasciculate (bundle) in the proximal part of the wing (Figure 3). Photos show that when the axons gradually fasciculated, the fluorescence in the cell bodies tended to disappear. By day 5, labeled cell bodies are absent, and there are straight labeled lines visible that correspond to axonal bundles. Axonal ‘pathfinding’ with irregularities such as hairpin structures and zigzag lines were also observed (Figure 3). This might be a consequence of the stress provoked by heat shock in these experiments. Taken together, these results demonstrate that the timing of neuronal development in the adult *Drosophila* wing can be extended and is capable of plasticity.

### Induced mitotic recombination reveals the birth of neuronal cells in the adult wing

**Figure 4:**
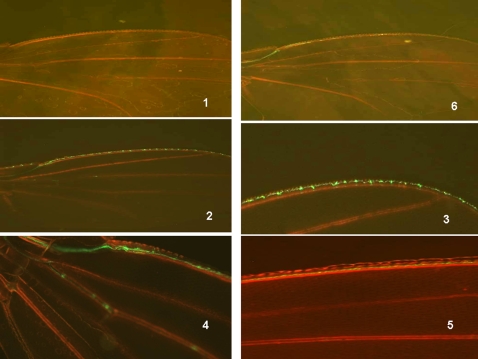
Recombination induced at the adult emergence stage: time course analysis of fluorescence in the corresponding wing. Progenies of the A+B cross (see *Materials and Methods*) were heat-shocked after adult emergence and wings were analyzed for GFP fluorescence at the indicated times. (1) Control heat shock at t = 0. (2,3) 5 hours after heat shock, (3) is a higher magnification of (2). (4) one day (5) five days and (6) three weeks after heat shock. See photo 6/figure 1 for orientation and dimensions.

We heat-shocked the progeny at the adult emergence stage in our model system and then analyzed the time course of the resulting fluorescence (Figure 4). A similar pattern of labeling as previously seen was obtained, except that the fluorescence was generally weaker than that generated by heat shock at the late third instar larva (or early pupa). The emergence of labeled cell bodies appeared first with weakly-labeled neuronal processes, and then a single straight axonal bundle was constantly seen. This fluorescence tends to disappear as the fly ages (only residual fluorescence is evident after 3 weeks). This strongly suggests that some neuronal cells are “born” in the emerged adult wing.

**Figure 5:**
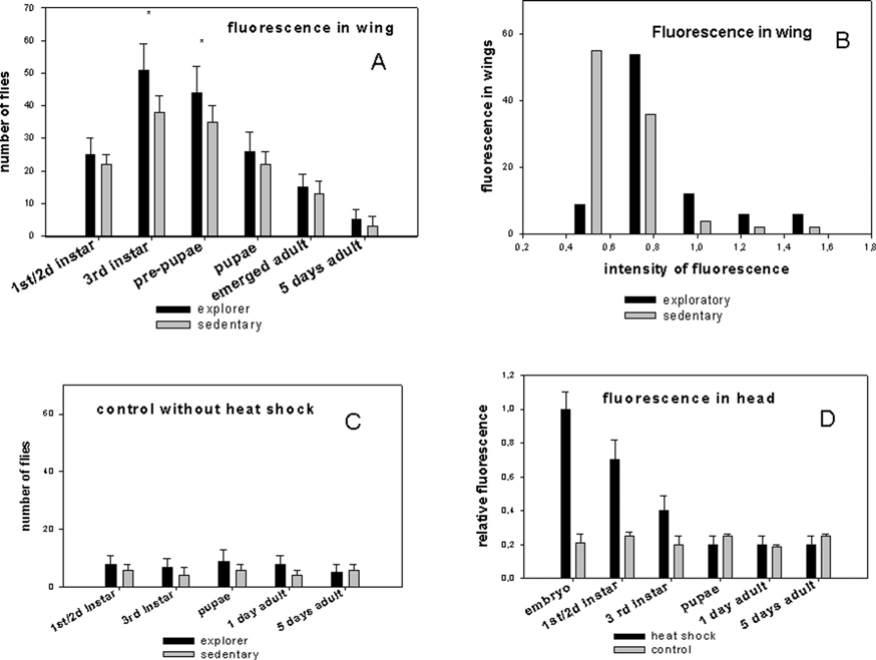
Measurement of adult wing fluorescence induced by recombination at different stages of the *Drosophila* life cycle using the *MARCM* system: influence of larval behavior. Graphs showing the comparative levels of fluorescence in two behavioral phenotypes: the exploratory and sedentary behaviors. The progenies of both phenotypes from the A+B cross of the *MARCM* system were separated at the third instar larval stage as indicated in the Methods. A heat-shock procedure was then carried out at different stages of the *Drosophila* life cycle. (A) Wings of 5 day old female flies (100 for each heat shock procedure) were dissected and analyzed for fluorescence. Bars are the mean +/−SD, n = 3, * P<0.01 (Student's t test). (B) Wings from 100 female flies presenting exploratory or sedentary behavior as larvae were analyzed using the five categories of fluorescence intensity arbitrarily set against standards. (C) Controls without heat-shock are shown. (D) A comparative measure of the total fluorescence in the adult heads from *MARCM* progenies (5 day old females flies, 100) corresponding to heat-shock treatments at different stages is represented.

The induction of mitotic recombination at different developmental stages confirms that the SOP originates from the late third instar and early pupal stages, but that the maturation process continues after the adults emerge from the pupae (Figure 5). The application of heat shock at the first and second instar larval stages resulted in substantial fluorescence in the wing, probably because some cells in the neuroectoderm, capable of generating an SOP, have already undergone recombination.

### The variability of adult wing neurogenesis in *Drosophila* is linked to a natural behavior polymorphism

We next investigated whether behavioral differences between larvae might account for the variations in neurogenesis in the wing at the adult stage. We utilized the well known natural polymorphism in *Drosophila* of larval foraging behavior to roughly establish two populations, one that remains on an ideal yeast food niche (sedentary), and one in which the flies migrate to ‘assess’ their environment (exploratory). This natural polymorphism seems to be preserved in the MARCM(A*B) progeny despite the use of the constructs allowing recombination. When flipase was induced in very late third instar larvae (the beginning of pupation), the number of flies showing fluorescence in the wing above an arbitrary threshold was higher for the exploratory larvae (Figure 5). We confirmed that flies originating from the exploratory larvae showed quantitatively more measured fluorescence compared with flies originating from sedentary larvae (Figure 5). However, only minor differences between the two behavioral categories were observed when the flipase enzyme was induced during the early larval stages or at the adult stage.

**Figure 6:**
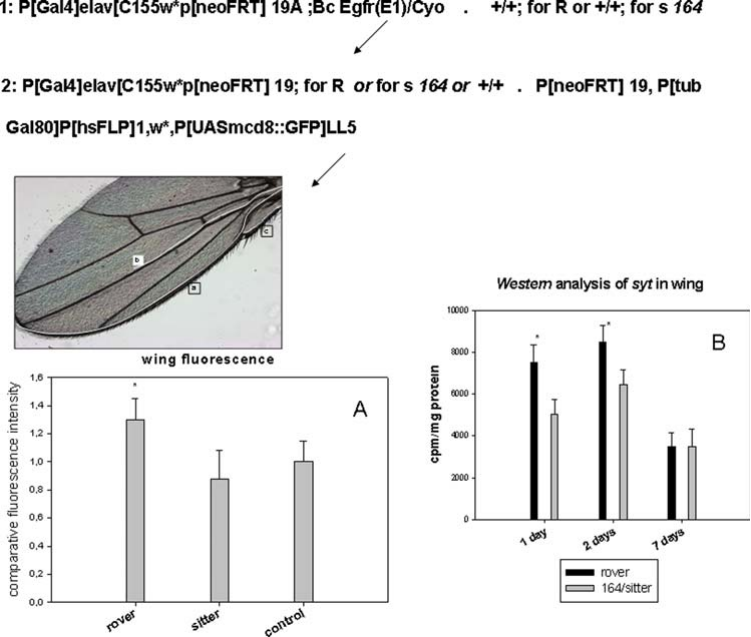
Quantification of fluorescence punctua in *MARCM* system progenies bearing the *Rover* or *sitter* alleles. *Rover* and *sitter* alleles were placed in the genetic background of one parent of the *MARCM* system as shown in the indicated scheme. Genetically modified A was crossed with B and progenies were then submitted to heat shock treatments at the late third instar larval stage. This procedure allows us to obtain a fluorescent *Rover* phenotype (*Rover* is dominant) and/or an enrichment of the fluorescent *sitter* phenotype. The control is the non-selected (total) progenies of the *MARCM* system. The three wing “pioneer tracts” are indicated in the representative image of the adult wing. (A)The fluorescence intensities were quantified in 3 days old wings from 100 females (see Methods). (B)The dosage of *synaptotagmin* molecules in 100 female wings strictly *Rover* or *sitter* strains is shown (*bottom right*). Differences between the two variants fade by day 7. Values represent the mean +/−SE, n = 3, * p<0,01 (Student test). Punctua were also counted in the costal nerve in one day old wings: control 63+/−5 Rover phenotype 68+/−5 and sitter phenotype 54+/−7, n = 20, * p<0,01

We also placed the homozygous alleles *Rover* or *sitter* (*164*) (*Rover* is dominant over *sitter* [29], [30]) in one parent of the *MARCM* system so that the progeny were either *Rover,* or enriched in the *sitter* allele. Wing analysis showed again that the *Rover* allele confers slightly more fluorescent signals and more anti-synaptotagmin immunoreactivity than the *sitter* allele (Figure 6). The number of fluorescent punctua in the wing margin after emergence is also greater in *Rover* than in *sitter* (legend in Figure 6). Moreover, the number of stout bristles in adult flies (mechanoreceptors) was compared and a small variability between individuals of the same sex was constantly observed in different genotypes but no significant variation was observed between explorer and sedentary larvae at adulthood (see Figure S5). This may indirectly indicate tiny stochastic differences in the number of mechanoreceptors.

**Figure 7:**
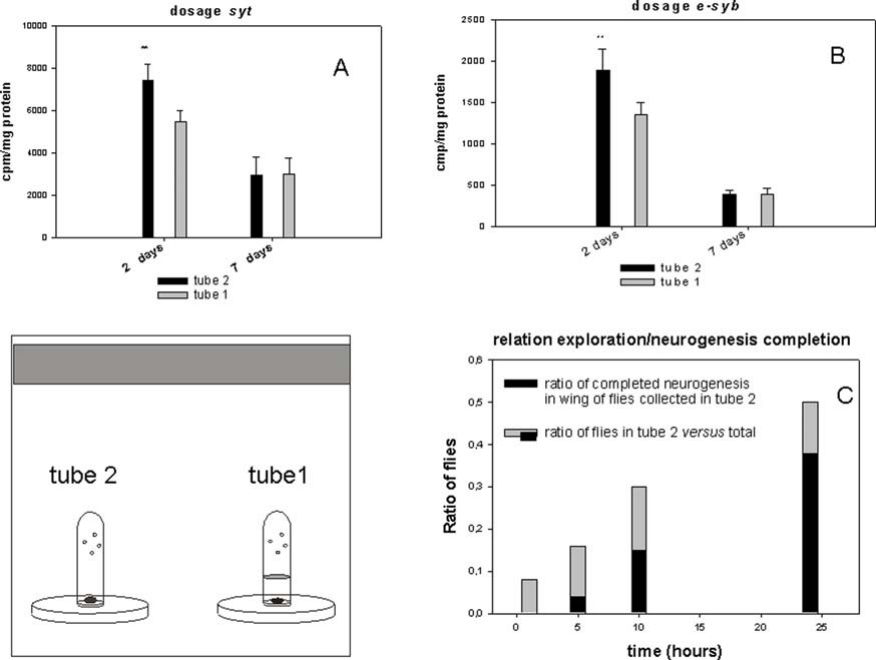
Dosage of *syt* and *syb* in adult wings from the exploratory and sedentary behavioral categories. (A and B): Same aged pupae (100) were placed in tube 1 with food (fixed around the disc). The flies in tube 2 with food (explorers) and tube 1 (sedentary) were collected two days after adult emergence. The wings were then cut off and analyzed by western blotting using anti *syt* or anti *syb* antibodies and protein A labeled with I^125^ (Bolton Hunter labeling, Amersham). Explorers showed significantly more labeling on day 2 (**p<0.01,bar are the mean +/−SD, n = 5) for these two molecules but these differences had faded by day 7. (C): individual pupae were placed in tube 1 without food. The emerged flies were counted in the tube 2 with food at the indicated times. The completion of neurogenesis was evaluated as a continuous fluorescent line corresponding to the nerve of the anterior wing margin. 25 *syt-GFP* flies were tested one by one and the graph represents the ratio of flies found in tube 2 *versus* total pupae and the proportion of achieved wing neurogenesis in those flies.

Because some expressed alleles of the *for* gene induce fidelity to the site of birth, whereas others induce a predilection to explore new habitats and engage in foraging [31], the differences in neurogenesis in the young adult wings Cantonese-S (*C-S*) was examined between these two behavioral populations i.e. flies (*C-S*) showing fidelity to their birth site and flies (*C-S*) flying away to find an alternative food spot. The anti synaptotagmin (*syt* ) and anti synaptobrevin (*e-syb*) immunoreactivity levels (two components of the neurosecretory vesicles measured as synaptic markers) were stronger in the two day old explorer adult wings (*C-S*) compared with the sedentary adult wing (*C-S*) (Figure 7). We show also that the completion of wing neurogenesis is correlated to exploration skills (Figure 7).

### The population density influences adult wing neurogenesis

The wing neuronal maturation of newborn *Drosophila* adults of the same genotype was examined to verify whether transient and non-heritable variants might be recruited from an undecided fate after adult emergence. This might also suggest that stochastically-generated variants in a limited window of time after adult birth constitute a natural transient polymorphism repertoire. We reasoned that this scenario, if verified, would facilitate a better adaptive response to the environment, including dispersion and consequently lower population density in crowdy niche where food ressources become limited to sustain demographic expansion.

**Figure 8:**
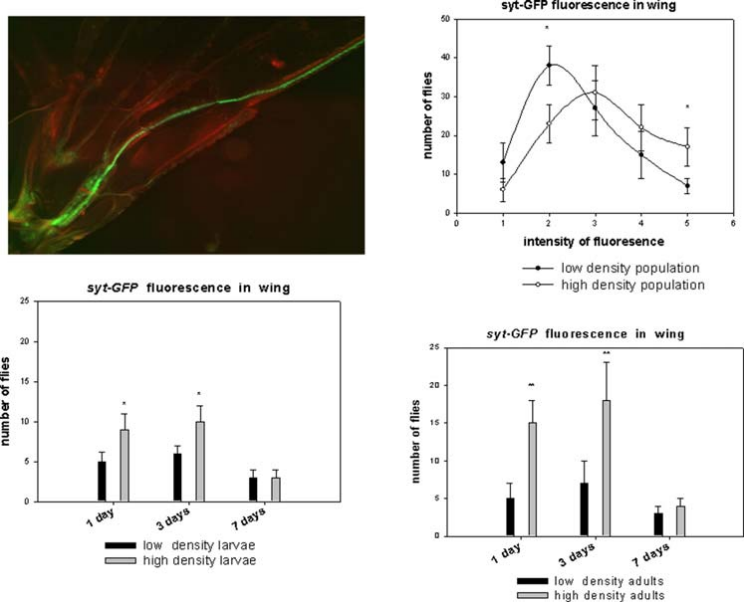
Analysis of the effects of population density on wing fluorescence using the *syt-GFP* construct. Fluorescence in adult *Drosophila* wings was analyzed in 2 day old female flies. Two groups of animals (both males and females) from the same larval conditions were kept at a high density (100 individuals in 20 ml air volume) and low density (10 flies in 200 ml air volume) after their emergence. At this stage, fluorescence shows stochastic variation. Fluorescence intensities equal to or above the levels shown in the photograph were determined from a total of 100 females for each experiment. The values shown are the mean +/−SE (n = 3). The same experiment was carried out with high density larvae (100 in 5 ml food volume) *versus* low density larvae (10 in 5 ml food volume). In this case the population densities of the adults were kept identical (100 in 200 ml volume) and the values are the mean +/−SE (n = 3) (* p<0.01, **p<0.001,*Student test*). Two day old flies (females) from three generations reared in high density or low density conditions were also analyzed over the full range of fluorescence (determined as 5 categories). Values are the mean +/−SE (n = 5 and *p<0.01). The five categories were analyzed in the context of contingency tables: Chi square value 12.0296 for a Prob =  0.0171 and DF: 4 (Fisher's exact test: table probability (P) 1.914E-06 and Pr< = P 0.0172).

This prompted us to analyze whether the larval or adult population densities could influence wing fluorescence. An alternative strategy was used for this analysis which involves a transgenic fly bearing the *GFP-syt* construct. This system was employed to overcome the risks of any side effects provoked by the heat shock step in the induced recombination procedure. The newly emerged homozygous female adults *GFP-syt* showed stochastic variations in their fluorescence levels as expected (see Figure S4). We then investigated whether a directional influence caused by environmental factors might modify wing sensory cell development. The breeding conditions in laboratory allowed us to control the larval and/or adult densities. A high density of adults or larvae significantly up-regulated the fluorescence patterns in the wing during the early stages of adulthood. After three days, the labeling tended to become weaker and no differences could eventually be detected among the animals (Figure 8). On the other hand, the *Rover* allele conferred transiently more *syt* immunoreactivity in wings of young adults compared with *sitter* when the flies were raised at a high density (see Table S1).

### Adult neurogenesis in *Drosophila* as a ‘time window’ for generating non-heritable variants in a fluctuating environment

**Figure 9:**
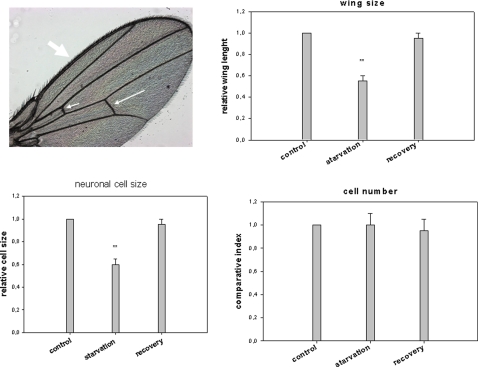
Analysis of sensory neurons in the small size adult wing generated by poor nutritional conditions. The *dnc* mutant is very sensitive to food conditions and generates proportioned but small bodies when the larvae are subjected to partial starvation. These tiny flies were placed in optimal food conditions after their emergence and showed full flight capabilities. The third generation was analyzed and showed phenotypic reversal. The number of mechanoreceptor neurons was deduced from the number of stout bristles. The cell number index was deduced by counting the number of hairs in the cross vein (small and median arrow) and the stout bristles along the wing margin.The relative cell size was the number of small hairs on the surface of the *Drosophila* wing each corresponds to a single cell divided by the length of the cross vein. Bar are the mean+/−SD, n = 10 (**p<0.01, *Student test*).

**Figure 10:**
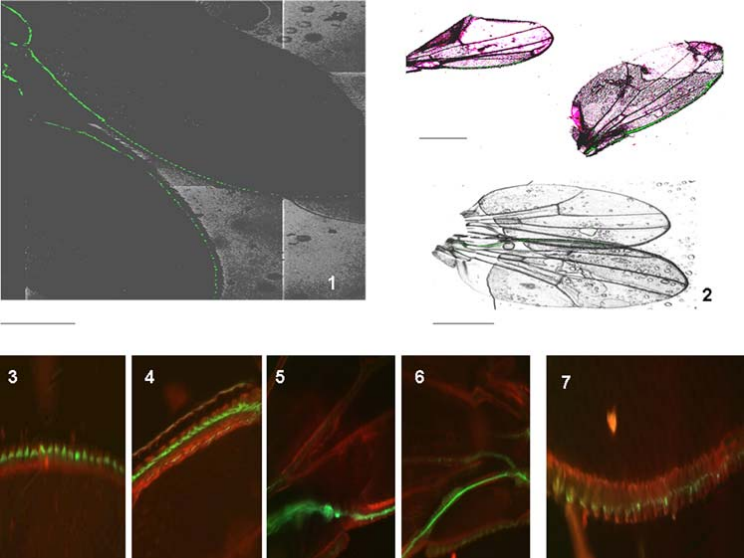
Analysis of neurogenesis in the adult wing from larvae subjected to starvation conditions. Larvae were subjected to starvation conditions (pupae were harvested from a 1 month old vial of stock or alternatively early third instar larvae were placed on agar/water medium) and the emerged adults were placed in optimal food conditions. This generates a normally proportioned small sized adult fly. (1) (dark photo): fluorescence in two day old female wings of the *syt- GFP* strain: (top) wing *syt*-*GFP* control and (bottom) wing *syt*-*GFP* from starved larva. These two wings are represented in the bottom right image in panel 2. Note the smaller size of the top wing (larva submitted to starvation). (2, *top*) : two day old female *syt-GFP* wings (a small wing from a starved larva and a control). Note that adult neurogenesis adapts to the smaller wing size. Two day old (3,5) and three day old (4,6) adult wings from starved *syt-GFP* larvae. An adult wing from a *syt-GFP* starved larva immediately after emergence is shown in (7). Note the delayed and chaotic, but preserved adult wing neurogenesis in the starved animals once optimal food conditions are returned after emergence. These photographs are representative of most of the reduced size animals. See figure 1 photo 6 for dimensions and orientation.

We analyzed how the nutritional conditions of the larvae might influence adult wing neurogenesis. It has been well documented in *Drosophila* larvae that defects in the insulin transduction pathway and/or starvation results in proportioned but smaller adult flies [41]. In our current experiments, when the mutant *dnc* (phosphodiesterase) was reared in a food-poor environment, the adults had smaller body and wing sizes (up to a 45% reduction). Wing analysis of these flies showed that this is mostly due to a decrease in cell size as the cell number seemed to be little affected (Figure 9). These definitive tiny flies retained a full flight capability when the newly-emerged adults were placed on an optimal food source. In addition, although their fecundity was reduced, these mutant flies were able to rescue their original body dimensions, including both wing and cell sizes, after two or three generations (Figure 9). The tiny newborn *syt-GFP* flies obtained after larval starvation also showed the ability to recover their wing neurogenesis when they were placed back on optimal food conditions. However, this neurogenesis appeared to be more chaotic and delayed compared with the controls (well fed larvae) (Figure 10). Wing neurogenesis restarts once optimal food conditions return, but develops according to the reduced dimensions imposed by the sizes of the pupae. We speculate that this incomplete neuronal architecture that follows the emergence of the adult flies from their pupae constitutes a ‘second chance’ mechanism that can overcome drastically unfavorable food conditions that exist during the larval stages.

### A unilateral lesion of the anterior wing margin nerve abolishes exploratory skills

**Figure 11:**
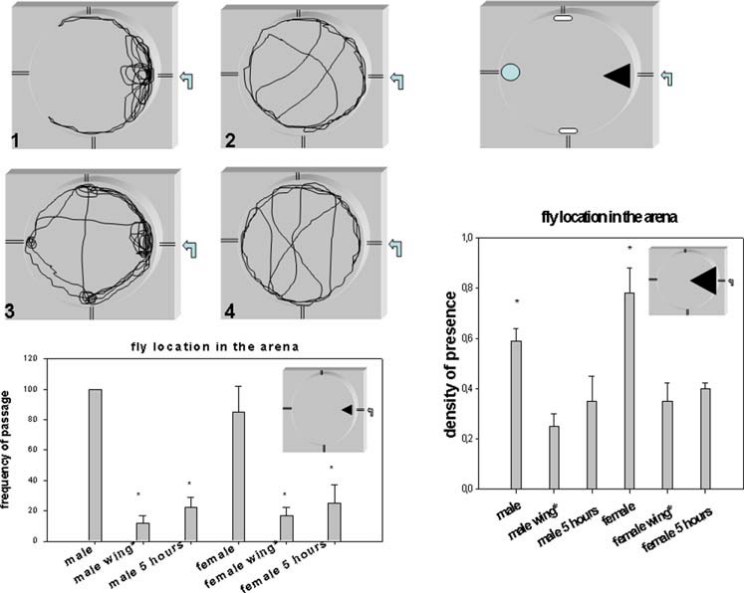
Exploration analysis of flies after unilateral lesion of the wing costal nerve and after emergence from pupa. (1–4) Flies were placed individually in an arena designed so that they can walk but not fly. A source of odorants (ethanol/apple juice, 1/3) is injected by a push syringe at 1ml/min (arrow) and the exploration of flies is monitored by a camera connected to software to enable analysis. A typical representative trajectory is shown for female (1,2) and male flies (3,4) (5 day old) with (2,4)or without (1,3) an unilateral wing costal nerve lesion (lesion is indicated as male wing* and female wing* in graphs). Four “check point” landmarks were used for the counting of fly passages (see *top righ*t , black triangle, blue circle and white oblong shape). *Bottom graph:* comparative frequency of passages in the black triangle landmark. The time for 100 passages for a male was used a time period to count the passages on the others flies. Bars are the mean +/−SE Student t test analysis; *p<0.001, n = 10. *Right graph*: proportion of time spent in black triangle for the indicated flies. The experiment was carried out on a 15 minutes period and flies were tested one by one. Bars are the mean +/−SE *Student t test analysis; *p<0.001, n = 20. Five day old flies with or without a wing nerve lesion and 5 hour old young flies were tested as indicated in the graphs.

We designed a system to analyze exploratory skills in an experimental arena where flies are prevented from flying using physical constraints. Flies therefore explore by walking. The experimental design and our results are summarized in Figure 11. The attractive combination of ethanol and grape juice odorants injected in the arena induced strong exploratory behaviour in individually tested flies. A behavioural sexual dimorphism was also observed; the males were very active and explored widely whereas the females were less active and only investigated the nearby origin of the odorant source. In both sexes, the typical pattern of trajectory was drastically perturbed after wing nerve lesion. Interestingly, the young flies with an unfinished wing neuronal wiring produced the same type of pattern as the flies with a wing nerve lesion. These data suggest that the wing neuronal architecture plays a role in space exploration and in navigation through odorant gradients. This might thus provide a hypothesis for a wing chemoreceptor function.

### Conclusions

In our present study, evidence for neurogenesis in the adult *Drosophila* wing is presented. This has not been previously elucidated for this structure but is a well documented phenomenon in the CNS of both adult insects and mammals [37]–[39], [42]–[46]. Our present data show that cell division in the adult wing is continued to complete the sensory neuronal architecture. The reason why this has not been reported previously is likely due to the extreme resistance of the cuticle to covalent bond breakage or dissolution by chemical agents or proteases. These technical difficulties have also greatly impaired immunohistological analysis of these structures. The *MARCM* system, however, allowed us to generate neuron-specific fluorescence under restrictive conditions i.e. a recombination event is required in the dividing precursor. By inducing this recombination at any stage of the *Drosophila* life cycle, we can track the point at which a cell division event occurs to produce a mature neuron. This approach turned out to be a powerful method for elucidating certain cellular events in the wing that have not been previously described.

By using this genetic method we were able to highlight previously unexpected steps during cell division in *Drosophila*. According to what has been revealed previously regarding the SOP lineage, fluorescence is expected to “pass” from a progenitor cell to one daughter cell only, but not to the sibling cell. In such instances, we would obtain only one labeled cell in the sensilla, regardless of its developmental stage. However, we detected distinct spots containing one, two or four labeled cells in campaniform sensilla of the same wing on the third vein. Because numerous papers assert that sensilla contain only one neuron, our data argues in favor of transiently expressed *elav* in non-neuronal cells. In the external fork of the anterior wing margin, clusters with two or four fluorescent cells were observed, which is in accordance with neuronal division in multiple innervated chemoreceptors. To distinguish between different scenarios will prove difficult until reliable markers are established that can discriminate between these cell identities.

Early reports have shown a first wave of axonogenesis within 1 or 2 hours after the onset of metamorphosis from neuronal cells born before pupariation [14]. A second wave has been described to arrive 12 hours later, and by 16 hours the nerve wing pattern is established [14]. This two steps process has been confirmed also for the campaniform sensilla on the third vein [47]. Our current data, however, seem to suggest the existence of extra waves of neuronal cell birth and axonogenesis after the fly emerges from pupae.

Many functions of wing sensory receptors (mechano- and chemoreceptors) are still poorly understood. Hypothetical roles were investigated in conditions where physical constraints prevented flight (arena system). When gradients of attractive odorants are generated inside such an arena, the exploratory mode was stereotyped and revealed a sexual dimorphism. Interestingly, the unilateral wing nerve lesion that was induced in 5 day old flies was found to cause a behavioural phenotype similar to immature adults (5 hours old). These results unambiguously reveal a role for sensory cells in the wing which is not limited to the mechanical control of flight. It is well known that flies spend considerable time brushing their wings and abdomen with their legs, which might stimulate some types of mechanoreceptors. Moreover, the hypothesis that molecules (volatile or not) might be spread mechanically to facilitate their binding to the taste like organs on wing is reinforced by the observation that “brooming” precedes exploration. This raises the fascinating question of what the behavioural impacts of the wing mechanoreceptors and contact chemoreceptors are when they are activated simultaneously and send coincident inputs via the same nerve.

We observed also a good match between the time course of male sexual maturity (between 2 and 3 days as reported elsewhere [48]–[50]) and the wing neurogenesis described in this report. This argues in favour of the hypothesis that the wingbeat frequency during courtship singing might be partially under the control of some sensory stimuli (contact or chemical) conveyed by wing nerves.

On the other hand, the general current belief is that cuticular structures (socket and hair) are completed at the emergence of the adult fly. The trichogen and tormogen cells constitutively found in the sensilla are known to be structured at the end of pupal stage and we show evidence that at this stage the neurogenesis of multiple innervated chemoreceptors underlying the gustatory sensilla are still under development. This delay in the neuronal architecture strongly supports the scenario that developmental chemosensory processing is awaiting epigenetic signals as suggested previously [51]. Specific plasticities for each wing system such as courtship singing, contact perception, chemical recognition by taste receptors and/or odorant binding by OBPs might be disconnected from each other. However, it is intuitive that mechanoreceptors should not be dependent on the local environment.

Many researchers have observed over a long period of time that there are small variations in the number of stout bristles in the *Drosophila* wing between male and female populations. We report herein that such variations also occur within the same genotype. Because these hairs constitute pierced tubes that shelter the dendrites of mechanoreceptors and form an intrinsic part of this sensory structure, we might indirectly assume that the number of mechanoreceptors slightly varies with the number of bristles. The fluorescent signals were found to fade as the animals aged, so we were not able to determine the final number of mechano/chemoreceptors at five days after adult emergence.

Phenotypic plasticity of the wing size and shape of *Drosophila* bred over a range of viable temperatures has been reported [22], [24]. Briefly, the wing size decreases when the temperature increases and at the opposite wing size increases in colder temperatures. It is generally believed that environmental conditions, such as nutrition, contribute to morphological variation between individuals by affecting body size or other morphological traits that are compatible with viable animals [22], [24]. The range of these modifications is also strictly limited, and defines rules for allometric plasticity in the context of environmental fluctuations [22]. These observations clearly show that epigenetic factors affect wing morphology and, consequently, wing neurogenesis. In this report, we describe that poor nutritional conditions generate non-heritable variants with a preserved adult neurogenic capacity which could be reactivated once optimal conditions return. However, this is far removed of well established discontinuous polyphenism that had been shown in species like *A. pisum* and that generates alternative and distinct morphotypes dependent upon the environment [52]. The late neurogenesis described in our present study seems to be an adaptive mechanism and a continuous ‘reaction norm’ to enable survival in a fluctuating environment.

On the other hand, our data show that the well characterized binary natural behavior polymorphism *Rover/sitter* affects multiple downstream genes and cellular systems. *Rover/sitter* is controlled by frequency dependent alleles of the gene *for* and its bimodal distribution suggests that the two groups (exploratory and sedentary) differ in the range of phenotypic plasticity that they are capable of manifesting when exposed to the same environmental constraints (see Table S1).

Furthermore, breeding conditions were experimentally manipulated in order to evaluate whether variability in neurogenesis might be directional, depending on the adult fly or larval population densities. Results suggest that demographic constraints confer a qualitative advantage in adult wing neurogenesis that likely enables exploration to be performed more efficiently at early stage of adulthood.

Selection should favor genetic/epigenetic intertwined mechanisms that adapt better individuals to adversity and/or hostility in unpredictable environments. Consistent with previous reports [23]–[26], 53, 54, our current study shows that the *Drosophila* genome is thus capable of producing subtle variants in a limited time window after adult birth and that this phenomenon is under the control of environmental factors to select the best fitness for the actual conditions.

Our present data also argue in favor of a non-synchronized process of adult wing neurogenesis after emergence and raise the question of the role of environmental cues in shaping appropriate variants. The genetic background selected by the prevailing ecological conditions might work in combination with the window of the developmental plasticity that we reveal in our present experiments. This strongly suggests that incomplete neurogenesis is a target for epigenetic regulation to recruit variants under control of environmental factors.

## Methods

### Experimental procedures

#### Mitotic recombination

The *Drosophila* flies used in this study were obtained from the Bloomington Center, Indiana University US and were A: P(GawB)elav C155 ,w, P[neoFRT] 19A, and B: P[neoFRT] 19 A, P(tubP-gal80)LL1, P[hsFLP]1,w'; P[UAs-mCD8::GFP]LL5 flies were crossed and the progenies were heat-shocked 3 times for 15 min at 37°C and kept for 15 min at room temperature between each heat treatment. These strains were kindly donated by the Liqun Luo laboratory and have been described previously [40]. The constructs are on the X chromosome, which implies that recombination induced by flipase occurs in females. Wings were cut at different stages, mounted on a glass slide with a drop of water and fixed with a cover slip. Samples were analyzed by fluorescence spectrophotometry at the excitation/absorption wavelengths recommended by manufacturer for GFP detection and quantification (Cary 300, Varian).

The flipase enzyme expressed under the heat-shock promoter exchanges arms after recognition of the FRT motifs. This strategy dissociates the inhibitor (Gal 80) from the activator transcription factor (Gal 4) so that the fluorescent axonal marker is expressed only in one daughter cell [40]. The constructs in the first chromosome used for recombination are shown. These strains are available at the *Bloomington Center* as the *MARCM* system. When the arms are exchanged during mitosis, the inhibitor of Gal 4-mediated transcription (Gal 80) and the activator of its transcription (Gal 4) become physically separated, the consequence of which is that each transgene will be expressed in one or the other daughter cell after recombination. In the absence of recombination, the co-presence of Gal 4 and Gal 80 blocks the transcription of mCD8:GFP, a neuron membrane targeted marker. When recombination takes place, mCD8:GFP is expressed in one daughter cell. This system allows the detection of dendritic and axonal projections, and the cell body of neurons also, but only when a recombination event occurs in a progenitor cell. Moreover, Gal 4 is under the control of the *Elav* promoter which is specifically activated in neurons, pIIb and pIIIb [40].

#### Genetic constructs used in this report

See Figure S1 for a schematic of the MARCM system.

P(GawB)elav C^155^ ,w, P[neoFRT] 19A; Bc[i] Egfr(E1)/Cyo was crossed with two strains: +/+; *Rover* or +/+; 164 (sitter allele) on the second chromosome. The resulting homozygous F_2_ *Rover* or *sitter* progeny were then crossed with the line P[neoFRT] 19 A, P(tubP-gal80)LL1, P[hsFLP]1,w'; P[UAs-mCD8::GFP]. The progeny of this cross were heat-shocked at the third instar larval stage using the same procedure described above. Adult females were analyzed as follows: the heads were removed with a razor and squashed to check the presence/absence of fluorescence. The positive samples were selected and the corresponding wings were analyzed as indicated above. *Rover* is dominant over *sitter* so one copy is sufficient to drive *Rover* behavior. Regarding *164* (sitter allele), this cross enriched for *sitter* behavior due to the fact that the P[UAs-mCD8::GFP] construct is on the second chromosome as the *for* gene (double heterozygous) and also that the P[UAs-mCD8::GFP] homozygous line shows a trend towards *sitter* behavior. In indicated experiment, the *dnc* mutant [55] very sensitive to food conditions was used to generate proportioned but small bodies when the larvae are subjected to partial starvation.

We also analyzed the fluorescent patterns in the wing using a line that expresses Gal4 and *syt.GFP* under the control of the *Elav* promoter (*Bloomington Center*). This line [UAS-*syt.GFP*: P[ GawB] elav[C155], P[UAS-syt.eGFP],w*] was used to follow the fluorescence of *synaptotagmin*, a constitutive component of synaptic vesicles, and its axonal transport from the cell body to the terminals in the thoracic structures.

#### Behavioral tests

Exploratory and sedentary behaviors were sorted at the third instar larval stage as indicated in references 28–30. When placed in optimal food patches, the larvae that escaped were deemed to be explorers and the larvae remaining on the patch to be sedentary.

Furthermore, in order to analyse the wing neuronal component in exploration and navigation, an arena was designed as indicated in figures. This system was designed to generate a gradient of odorants in a controlled manner inside the arena volume where flies are able to walk but not to fly. This arena is a circle of 30 cm diameter and 7 mm in depth. The plastic structure contains four channels smaller to the fly body size, one of which is connected to the odorants source. This structure is placed in sandwich between two glass plates. 5 ml of ethanol and grape juice (1/3) in a 30 ml glass syringe was used to generate odors. The air of the syringe connected to the arena by a capillary was injected using an automatic syringe pusher at 1 ml/minute to create a gradient. A camera linked to a computerized analysis system was placed above the arena in order to monitor the trajectories as described elsewhere [56]. Flies were tested as indicated in the figure legends one by one, male and female. For quantification, four landmarks as represented in figures were used to count the passages. The experiments were conducted for 15 minutes, beyond which time the arena starts to be saturated (flies starved for 2 hours before experiments). In the indicated experiments, the time necessary for 5 day old males (*C-S*) to perform 100 passages was used as a standard to determine the comparative number of passages for flies under conditions indicated in the figures.

To generate a unilateral lesion of the wing nerve, flies were immobilized under CO_2_ and a razor was used to cut the anterior wing margin as indicated elsewhere [7]. This procedure does not alter the life span of the flies [7].

#### Quantification of fluorescence in the adult wings and head extracts

Flies A and B were crossed and the resulting progeny were heated-shocked 3 times for 15 min at 37°C, each heat treatment being separated by a 15 min incubation at room temperature. At the third instar larval stage, exploratory and sedentary behaviors were analyzed and sorted. The heat shock procedure was carried out at the indicated period of the fly life cycle (when the heat-shock was delivered at the very late third instar stage, the exploratory and sedentary behaviors were sorted out first). At day 5, the heat-shock procedure was carried out a few hours prior to the fluorescence analysis. Regardless of the timing of the heat shock treatment, the wings of the flies were excised on day 5 with a razor and placed onto glass slides. An arbitrary level of fluorescence was determined as the background standard and wings above or equal to this level were counted. This analysis was carried out visually with a fluorescence microscope (Leica MZFLIII) (from 1 X to 10 X original magnification) and verified by quantification of fluorescence using a Cary spectrophotometer and multi well plate (one wing was placed in 50 ul of water in each well). Only females were considered for analysis.

As indicated in the figures, five categories for the intensity of fluorescence were also determined using fluorescent flies as standards. The wings were visually attributed to one of these 5 categories using a fluorescence microscope and this was verified by quantification with a Cary spectrophotometer. One hundred female flies were analyzed for each experiment and this trial was repeated 3 times.

Fluorescence was also analyzed in adult head extracts. The adult heads were removed with a razor and resuspended in a phosphate buffer (10 mM at pH 7.4) using a dual glass homogenizer. Because the fluorescent marker is a membrane hybrid molecule, the extract was sonicated for 1 min (Bioblok sonicator, power 30) in order to access the fluorescent probe. Fifty female flies were ground on day 5 in 1 ml buffer under the conditions described in the figure legends and the fluorescence values were measured for aliquots of 100 µl in a multi-well plate with a Cary spectrophotometer (Varian analytical instrument). This experiment was also repeated 3 times.

#### Dosage of synaptotagmin (*syt*) and synaptobrevin (*syb*) in adult wings from the exploratory and sedentary behavioral categories

Two identical tubes were placed in a chamber but the same aged pupae were placed only in tube 1 (see Figure 7). Equal amounts of yeast food were then placed inside tubes 1 and 2, which were pierced with holes to allow easy access to the flies. The flies in tube 2 (explorers) and tube 1 (sedentary) were collected two days after adult emergence of the pupae. The wings from 50 flies were cut off and ground in 100 µl SDS 1% and after brief centrifugation, the supernatant (50 µl) was analyzed by western blotting using anti *syt* or anti *syb* antibodies and protein A labeled with I^125^ (Bolton Hunter labeling, Amersham). The nitrocellose bands corresponding to the molecular weights were cut and counted in a Beckman *gamma* counter.

## Supporting Information

Figure S1 Genetic scheme for the mitotic recombination events that drive fluorescence in Drosophila neurons (constructs obtained from the Bloomington Center).
(0.06 MB TIF)

Figure S2 Control of fluorescence in the adult wing of the MARCM system progeny without heat shock. (a) and (b), the P [neoFRT]19A, P [tub-Gal80] LL1, P[hs FLP]1, w*; P[UAS-mCD8::GFP] strain with and without heat shock, respectively. When this strain is crossed with P [Gaw B] elav C155, w*,p[neoFRT] 19 A, the progenies bearing one copy of the two chromosomes do not show any substantial fluorescence signal without heat shock. However, we did observe very marginal fluorescence in the wing, likely due to a leaky hsp-flipase. These data constitute the controls for all the experiments described in this report. Also shown are representative images of wings from crosses without heat shock at adult emergence (c), and from 1 day (d) and 5 days old flies (e).
(0.96 MB TIF)

Figure S3 Heterogeneity of fluorescence in two day old adult wings generated by the MARCM system. (1-5): wings from a one day old female fly. Clusters of neuronal cells (chemoreceptors) are clearly evident. Some clusters show 5 to 6 cells (big arrow) or 2 cells (small arrow), although the mature sensilla are identical. We also see heterogeneous processes in the same wing margin (middle left) and observe variable fluorescent patterns between wings from different flies (top). (6,7): differences in the number of clusters in the fork from 2 day old wings (6 versus 3). (8): represents a higher magnification of the wing proximal portion.
(1.22 MB TIF)

Figure S4 Analysis of fluorescence in the P[ GawB] elav[C155], P[UAS-syt.eGFP],w* strain. Wings of two day old female flies show strong variations in the intensity and the pattern of labeling, which suggests that stochastic processes of sensory neuron maturation occur. syt. GFP is a hybrid molecule of synaptotagmin which is constitutively expressed in neurosecretory vesicles. The fluorescence we see is therefore linked to the synthesis of neurosecretory vesicles and their axonal transport from the cell body to the terminals in the thoracic structures. The signal patterns in these strains are very similar to those obtained using the recombination strategy. Panels 1–3 and 7–9 show the proximal part of the wing margin. Panels 4–5 show the distal part of the wing margin and 6 and 9 are controls without GFP.
(1.15 MB TIF)

Figure S5 Number of stout bristles in the wing margin from the cross vein to the distal extremity. Bristles were counted in females flies of different genotypes. The white oval area was counted from the cross vein to the distal part. Photographs above show significant differences between two female flies. We observed very small differences between the strains and we detect variability between individuals in each strain (see below): Rover : 9.28+/−0.275* (p<0.05 versus Cs) [from 8 to 12] sitter : 11.57+/−0.375 [from 10 to 14] Y2-2 : 9.4+/−0.5* (p<0.05 versus Cs) [from 8 to 11] dnc : 11.1+/−0.2 [from 10 to 13] rut : 11+/−0.2 [from 10 to 12]
(0.47 MB TIF)

Table S1 Relationship between population density and syt synthesis in the Drosophila wing: influence of the Rover/sitter background. Ratio between the syt levels in a high versus a low adult population density and in a high versus a low larval population density. Newborn adults were maintained at high density (100 flies per vial during the first two days) or low density (10 flies per vial) and/or 100 larvae (high density) versus 10 larvae (low density) per vial. The dosage of syt was determined using Bolton Hunter labeled protein A after gel electrophoresis of two day old wing extracts. Values represent the mean ratios of the syt levels in high versus low density populations for three determinations. *p<0.01 versus sitter, Student test). The control experiments followed the same protocol using head extracts and an anti HRP antibody (neuronal marker).
(0.02 MB DOC)
