# Supplementary figures and images for: Continued Neurogenesis in Adult Drosophila as a Mechanism for Recruiting Environmental Cue-Dependent Variants
Source: PLoS One. 2008 Jun 11;3(6):e2395. doi: 10.1371/journal.pone.0002395 (PMC2405948; doi:10.1371/journal.pone.0002395)

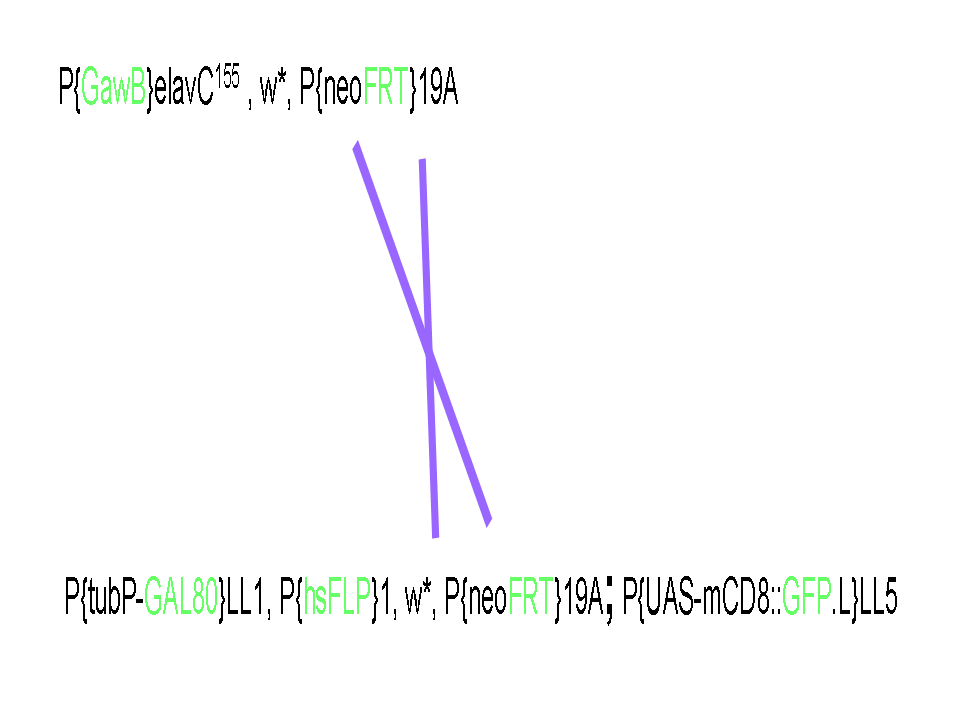

Supplement: Figure S1 — Genetic scheme for the mitotic recombination events that drive fluorescence in Drosophila neurons (constructs obtained from the Bloomington Center). (0.06 MB TIF) [file pone.0002395.s001.tif]

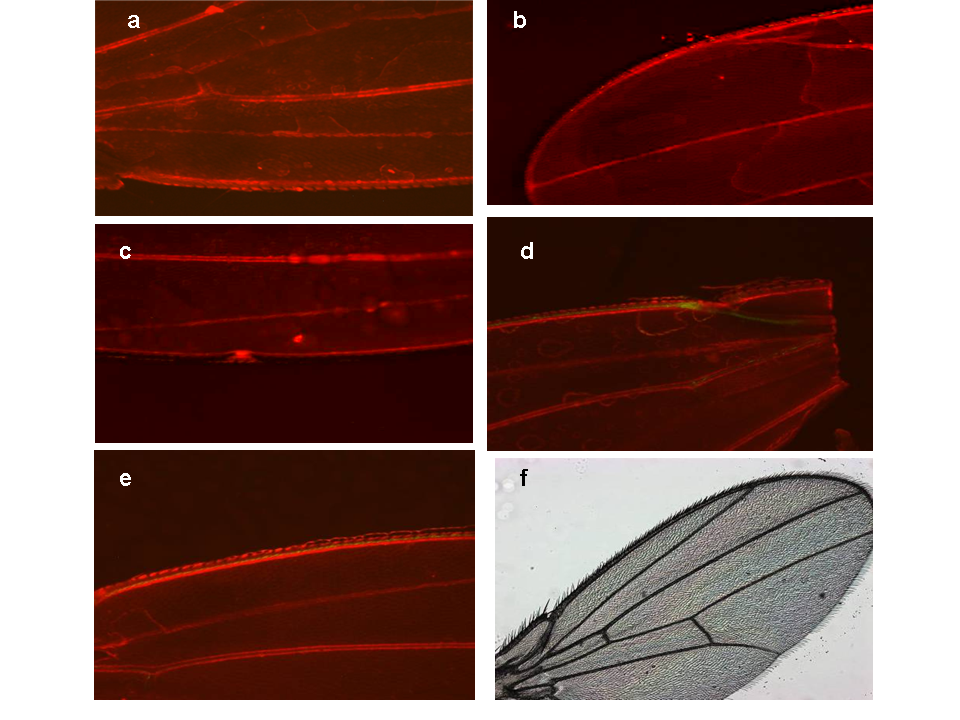

Supplement: Figure S2 — Control of fluorescence in the adult wing of the MARCM system progeny without heat shock. (a) and (b), the P [neoFRT]19A, P [tub-Gal80] LL1, P[hs FLP]1, w*; P[UAS-mCD8::GFP] strain with and without heat shock, respectively. When this strain is crossed with P [Gaw B] elav C155, w*,p[neoFRT] 19 A, the progenies bearing one copy of the two chromosomes do not show any substantial fluorescence signal without heat shock. However, we did observe very marginal fluorescence in the wing, likely due to a leaky hsp-flipase. These data constitute the controls for all the experiments described in this report. Also shown are representative images of wings from crosses without heat shock at adult emergence (c), and from 1 day (d) and 5 days old flies (e). (0.96 MB TIF) [file pone.0002395.s002.tif]

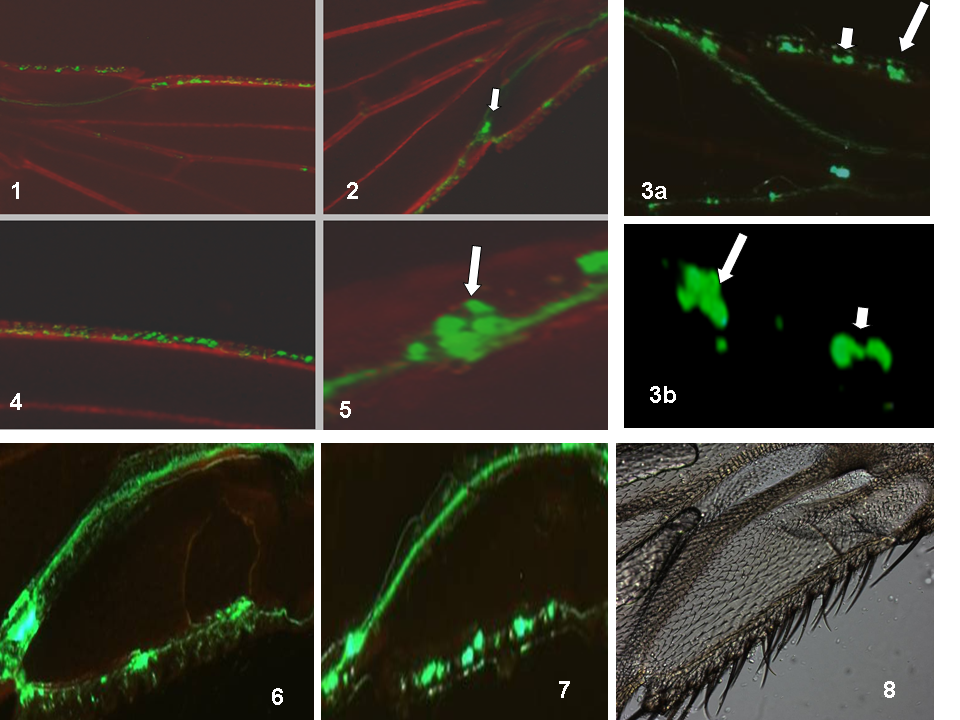

Supplement: Figure S3 — Heterogeneity of fluorescence in two day old adult wings generated by the MARCM system. (1-5): wings from a one day old female fly. Clusters of neuronal cells (chemoreceptors) are clearly evident. Some clusters show 5 to 6 cells (big arrow) or 2 cells (small arrow), although the mature sensilla are identical. We also see heterogeneous processes in the same wing margin (middle left) and observe variable fluorescent patterns between wings from different flies (top). (6,7): differences in the number of clusters in the fork from 2 day old wings (6 versus 3). (8): represents a higher magnification of the wing proximal portion. (1.22 MB TIF) [file pone.0002395.s003.tif]

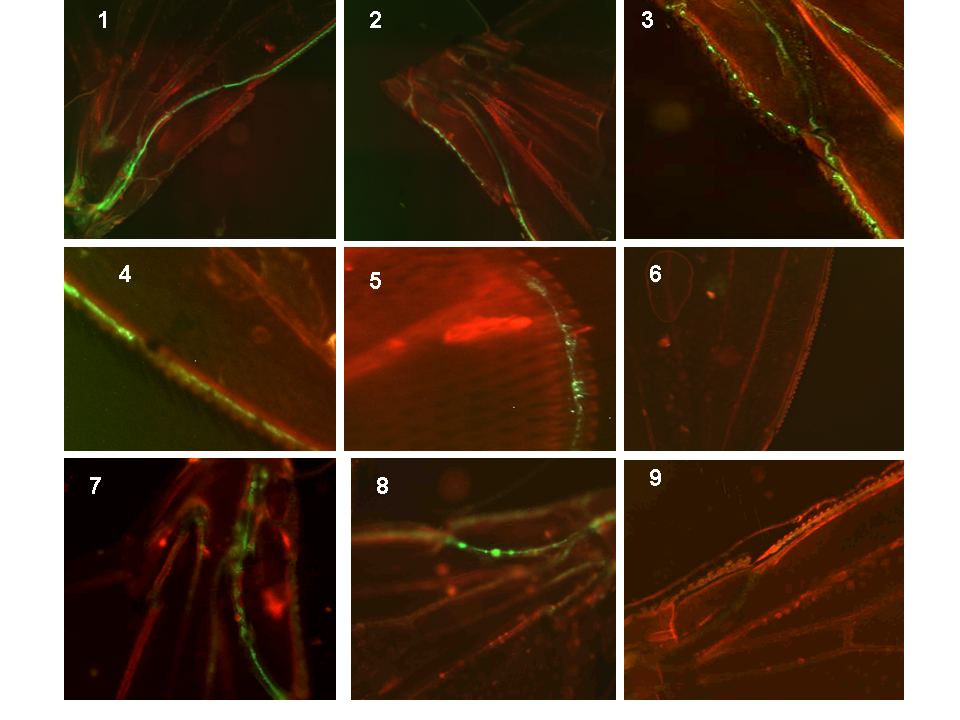

Supplement: Figure S4 — Analysis of fluorescence in the P[ GawB] elav[C155], P[UAS-syt.eGFP],w* strain. Wings of two day old female flies show strong variations in the intensity and the pattern of labeling, which suggests that stochastic processes of sensory neuron maturation occur. syt. GFP is a hybrid molecule of synaptotagmin which is constitutively expressed in neurosecretory vesicles. The fluorescence we see is therefore linked to the synthesis of neurosecretory vesicles and their axonal transport from the cell body to the terminals in the thoracic structures. The signal patterns in these strains are very similar to those obtained using the recombination strategy. Panels 1–3 and 7–9 show the proximal part of the wing margin. Panels 4–5 show the distal part of the wing margin and 6 and 9 are controls without GFP. (1.15 MB TIF) [file pone.0002395.s004.tif]

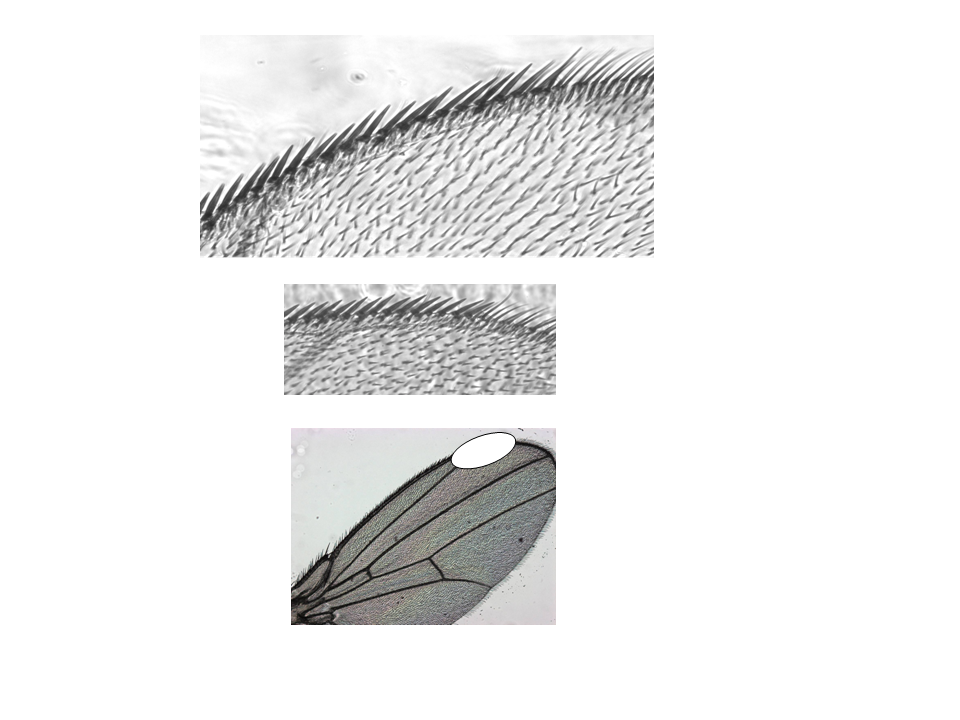

Supplement: Figure S5 — Number of stout bristles in the wing margin from the cross vein to the distal extremity. Bristles were counted in females flies of different genotypes. The white oval area was counted from the cross vein to the distal part. Photographs above show significant differences between two female flies. We observed very small differences between the strains and we detect variability between individuals in each strain (see below): Rover : 9.28+/−0.275* (p<0.05 versus Cs) [from 8 to 12] sitter : 11.57+/−0.375 [from 10 to 14] Y2-2 : 9.4+/−0.5* (p<0.05 versus Cs) [from 8 to 11] dnc : 11.1+/−0.2 [from 10 to 13] rut : 11+/−0.2 [from 10 to 12] (0.47 MB TIF) [file pone.0002395.s005.tif]
