# Supplementary material for: Continued Neurogenesis in Adult Drosophila as a Mechanism for Recruiting Environmental Cue-Dependent Variants
Source: PLoS One. 2008 Jun 11;3(6):e2395. doi: 10.1371/journal.pone.0002395 (PMC2405948; doi:10.1371/journal.pone.0002395)
Supplement: Table S1 — Relationship between population density and syt synthesis in the Drosophila wing: influence of the Rover/sitter background. Ratio between the syt levels in a high versus a low adult population density and in a high versus a low larval population density. Newborn adults were maintained at high density (100 flies per vial during the first two days) or low density (10 flies per vial) and/or 100 larvae (high density) versus 10 larvae (low density) per vial. The dosage of syt was determined using Bolton Hunter labeled protein A after gel electrophoresis of two day old wing extracts. Values represent the mean ratios of the syt levels in high versus low density populations for three determinations. *p<0.01 versus sitter, Student test). The control experiments followed the same protocol using head extracts and an anti HRP antibody (neuronal marker). (0.02 MB DOC) [file pone.0002395.s006.doc]

**Supplementary Table S1**

**high *versus* a low adult population density**

*Rover*: 1.38 +/- 0.3* *control HRP*: 1.05+/-0.5

*sitter (164)*: 1.05+/- 0.1 : 1.01+/-0.25

*C-S*: 1.25+/-0.15 : 1.05+/-0.5

**high *versus* a low larval population density**

*Rover*: 1.25+/-0.25* *control HRP*: 1.05+/-0.5

*sitter (164)*: 1.1+/-0.1 : 0.99+/- 0.25

*C-S*: 1.1+/-0.1 : 0.99+/-0.25
